# Multi-institutional study of nuclear KIFC1 as a biomarker of poor prognosis in African American women with triple-negative breast cancer

**DOI:** 10.1038/srep42289

**Published:** 2017-02-20

**Authors:** Angela Ogden, Chakravarthy Garlapati, Xiaoxian (Bill) Li, Ravi Chakra Turaga, Gabriela Oprea-Ilies, Nikita Wright, Shristi Bhattarai, Karuna Mittal, Ceyda Sönmez Wetherilt, Uma Krishnamurti, Michelle D. Reid, Mildred Jones, Meenakshi Gupta, Remus Osan, Sonal Pattni, Ansa Riaz, Sergey Klimov, Arundhati Rao, Guilherme Cantuaria, Padmashree C. G. Rida, Ritu Aneja

**Affiliations:** 1 Georgia State University, Department of Biology, Atlanta, GA, USA; 2 Emory University School of Medicine, Department of Pathology, Atlanta, GA, USA; 3 Northside Hospital Cancer Institute, Atlanta, GA, USA; 4 West Georgia Medical Center, Department of Pathology, LaGrange, GA, USA; 5 Georgia State University, Department of Mathematics and Statistics, Atlanta, GA, USA; 6 Scott and White Medical Center, BSWHealth, Temple, TX, USA; 7 Novazoi Theranostics, Rolling Hills Estates, CA, USA.

## Abstract

Nuclear KIFC1 (nKIFC1) predicts worse outcomes in breast cancer, but its prognostic value within racially distinct triple-negative breast cancer (TNBC) patients is unknown. Thus, nKIFC1 expression was assessed by immunohistochemistry in 163 African American (AA) and 144 White TNBC tissue microarrays (TMAs) pooled from four hospitals. nKIFC1 correlated significantly with Ki67 in White TNBCs but not in AA TNBCs, suggesting that nKIFC1 is not merely a surrogate for proliferation in AA TNBCs. High nKIFC1 weighted index (WI) was associated with significantly worse overall survival (OS), progression-free survival (PFS), and distant metastasis-free survival (DMFS) (Hazard Ratios [HRs] = 3.5, 3.1, and 3.8, respectively; P = 0.01, 0.009, and 0.007, respectively) in multivariable Cox models in AA TNBCs but not White TNBCs. Furthermore, KIFC1 knockdown more severely impaired migration in AA TNBC cells than White TNBC cells. Collectively, these data suggest that nKIFC1 WI an independent biomarker of poor prognosis in AA TNBC patients, potentially due to the necessity of KIFC1 for migration in AA TNBC cells.

KIFC1, a minus end-directed microtubule motor belonging to the C-terminal kinesin subfamily^1^, crosslinks and slides microtubules in mammalian meiotic and mitotic spindles, facilitating tight pole focusing^2^. This kinesin can also bind to and traffic early endocytic vesicles^3^ and DNA olignonucleotides^4^ along microtubules. Recently, attention has focused on KIFC1 due to its association with malignancy. High KIFC1 transcript levels in lung tumors predict increased risk of metastatic dissemination to the brain^5^. Primary breast tumors also overexpress KIFC1 as compared with matched normal breast tissue^6^, and TNBCs express higher KIFC1 than non-TNBCs^7^. nKIFC1 expression correlates with advanced tumor grade and worse OS and PFS in breast cancer^6^. KIFC1-overexpressing MDA-MB-231 and MDA-MB-468 TNBC cells exhibit enhanced survival compared with vector controls^8^. KIFC1 may contribute to apoptosis reluctance in TNBC because KIFC1 overexpression stabilizes survivin by decreasing its polyubiquitination in MDA-MB-231 cells^6^. KIFC1 promotes the survival of TNBC cells with supernumerary centrosomes, which rely on KIFC1 for clustering of supernumerary centrosomes, thereby facilitating chromosomal instability^9^,^10^. Indeed, *KIFC1* was identified as the top hit in a genome-wide Drosophila screen of centrosome clustering genes, and it is essential for clustering of supernumerary centrosomes and suppression of multipolar division of human cancer cell lines^9^.

AA women with breast cancer experience a more aggressive clinical course than White women with breast cancer partly due to the ~2–3-fold increased risk AA women have of developing TNBC, a subtype with a high risk of distant relapse and mortality; nonetheless, racial disparity may exist even within this subtype^11^,^12^. A critical need exists for validation of novel biomarkers to risk-stratify AA breast cancer patients because they experience higher breast cancer mortality than any other racial group, which might indicate that high-risk AA patients are not being identified as such using standard clinical prognostic tools and are thus not being prescribed sufficiently aggressive treatment. TNBC remains defined by what biomarkers it lacks, whereas non-TNBCs are defined by expression of hormone receptors and/or HER2, which can be targeted with specific inhibitors. Treatment guidelines in the US stratify TNBC patients for systemic adjuvant treatment based primarily on tumor staging. The association of nKIFC1 with triple-negative receptor status and worse clinical outcomes in breast cancer suggests that nKIFC1 drives aggressive breast cancer disease course and may potentially serve as a prognostic biomarker in TNBC, although its utility in specific racial groups is unclear. Given that AA women with breast cancer suffer a more aggressive disease course than White women, we hypothesized that KIFC1 would hold greater value as a prognostic biomarker in AA women with TNBC. Given the association between KIFC1 and brain metastases in lung cancer, we also hypothesized that KIFC1 is critical for TNBC cell migration. Herein, we analyze nKIFC1 expression by immunohistochemistry in race-annotated TNBC specimens to test its association with race, standard clinical prognostic factors, and clinical outcomes within racial groups and determine the effect of KIFC1 knockdown on migration and proliferation in White and AA TNBC cell lines.

## Results

**Figure 1:**
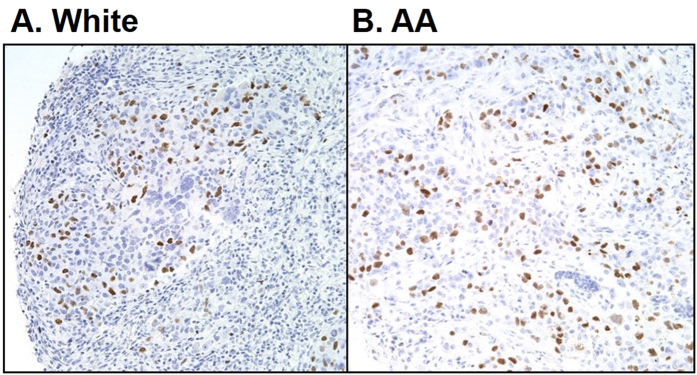
Representative images of nKIFC1 staining in (**A**) White and (**B**) African American (AA) triple-negative breast tumors. 20X objective.

We found that nKIFC1 WI was significantly higher in AA than White TNBCs (154.66 vs. 133.74, respectively, p = 0.036) (Fig. 1, representative staining). In Whites, nKIFC1 WI was significantly higher in grade 3 than grade 1 and 2 TNBCs (p = 0.035 and p = 0.001, respectively, per post-hoc comparison), whereas nKIFC1 WI did not significantly differ by grade in AA TNBCs (Table 1). nKIFC1 WI was significantly higher in high-stage AA TNBCs, whereas nKIFC1 WI did not significantly differ by stage in White TNBCs. In both races, nKIFC1 WI did not significantly differ by lymph node status, and nKIFC1 WI was not significantly correlated with tumor size or age at diagnosis.

Among a panel of 21 potential biomarkers, Ki67 WI alone was significantly correlated with nKIFC1 WI in White TNBCs (ρ = 0.65, p = 0.00076), whereas no significant correlations were found in AA TNBCs, suggesting that nKIFC1 is not merely a surrogate of any of these biomarkers in AA TNBCs.

nKIFC1 was not significantly associated with any survival outcome in simple Cox models (Table 2) or Kaplan-Meier analysis (Supplementary Figures 1–3). However, in multivariable analysis adjusting for tumor stage, age at diagnosis, chemotherapy, and hospital, high nKIFC1 WI was associated with >3 times worse OS, PFS, and DMFS in AA TNBCs (bootstrap p < 0.05 for all), whereas it did not significantly impact any survival outcome in White TNBCs.

**Figure 2:**
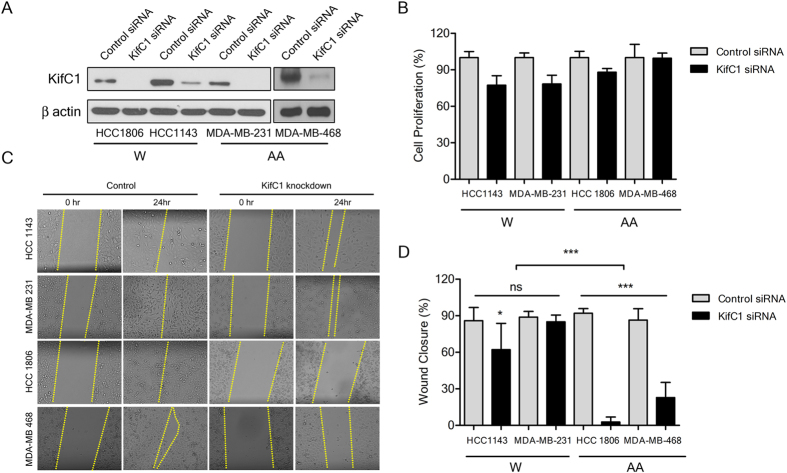
Impact of KIFC1 knockdown on proliferation and migration of White (W) (HCC1143 and MDA-MB-231) and African American (AA) (HCC1806 and MDA-MB-468) triple-negative breast cancer (TNBC) cell lines. (**A**) Western blots of KIFC1 from White and AA TNBC cell lines treated with control or KIFC1 siRNA. (**B**) Proliferation of White and AA TNBC cell lines following treatment with control or KIFC1 siRNA. (**C**) Micrographs from wound healing assays of White and AA TNBC cell lines treated with control or KIFC1 siRNA. (**D**) Percentage wound closure in White and AA TNBC cell lines following treatment with control or KIFC1 siRNA. *p < 0.01; ***p < 0.0001; ns = not significant.

To gain insights into why KIFC1 is prognostic in AA TNBCs but seemingly not White TNBCs, we performed proliferation and wound-healing assays with 2 AA and 2 White TNBC cell lines in which KIFC1 was transiently knocked down with siRNA (Fig. 2A). We found that KIFC1 knockdown did not significantly impact proliferation in any of the cell lines (Fig. 2B). By contrast, KIFC1 knockdown significantly impaired wound healing in one of the White TNBC cell lines and both AA TNBC cell lines; however, the percentage wound closure was more drastically reduced in AA TNBC cell lines (p < 0.0001) (Fig. 2C,D). These results suggest that AA TNBC cells more heavily rely on KIFC1 for migration than EA TNBC cells, which may partly underlie the differential prognostic importance of KIFC1 by race as we found in clinical TNBC specimens.

## Discussion

AA breast cancer patients are known to suffer increased cancer-related morbidity and mortality compared with all other racial groups even after adjusting for socioeconomic and lifestyle factors^13^,^14^,^15^. In particular, AA TNBC patients have a particularly dire prognosis, especially those with high-stage tumors. Given that AA women afflicted with breast cancer typically suffer from a much more aggressive disease course, it is critical to identify and validate biomarkers that could indicate differences in tumor biology between racial groups and serve as risk predictors that would help to stratify AA patients better and mitigate health disparity in disease outcomes.

In the clinic, establishment of triple-negative receptor status is a crucial factor determining treatment choice for patients because the absence of therapeutic targets in TNBC patients (a large fraction of whom are AA) leaves clinicians with no option but to resort to conventional chemotherapeutics that are non-discriminating and highly toxic. AA patients have also not benefited from the advances in breast cancer therapy as much as other patient groups, owing in large part to the high incidence of TNBC in this racial group. In our study, nKIFC1 was found to be a strong predictor of significantly worse survival outcomes in multivariable analysis in AA TNBCs. Our finding is valuable because it positions nKIFC1 not only as a critical racial disparity biomarker in TNBC but also as great potential therapeutic target. For the first time, AA patients whose tumors are triple-negative can be categorized based on what their tumors express, rather than what they lack (hormone receptors and HER2). Importantly, in AA TNBCs nKIFC1 WI does not significantly correlate with WIs of members a large panel of potential cancer biomarkers, suggesting it is not merely a surrogate for them and can offer novel prognostic information. It is remarkable that nKIFC1 significantly predicts survival outcomes in AA but not White TNBCs considering the extensive overlap in nKIFC1 scores between the two groups, since nKIFC1 was only slightly (albeit significantly) higher in AA than White TNBCs. This finding suggests that the differential prognostic value of nKIFC1 between these groups may arise from inherently different biology, possibly rooted in genetic ancestry, rather than substantially higher nKIFC1 levels in AA than White TNBCs. Our finding that AA TNBC cell lines are more reliant on KIFC1 for migration provides possible mechanistic insights into why nKIFC1 levels are prognostic in AA but not White TNBCs. AA TNBC cells with high nKIFC1 levels may more efficiently migrate and thereby metastasize distantly, conferring worse prognosis. Our failure to reject the null hypothesis that there is no difference between nKIFC1-low and high groups among White TNBC patients aligns with our finding that KIFC1 is not entirely necessary for the migration of White TNBC cells; however, we may have failed to reject the null not because no true difference exists between nKIFC1-low and high White TNBC groups, but rather because the difference is very small or perhaps because of sampling or other errors, given that one White TNBC cell line relied to a significant extent on KIFC1 for migration. Larger future studies may be able to lend more confidence to our findings regarding a seeming lack of prognostic value of nKIFC1 among White TNBCs.

Our study, which has unraveled nuclear accumulation of the kinesin motor protein KIFC1 as a novel biomarker for cancer’s unequal burden within the AA TNBC population, and which also demonstrates that KIFC1 is more important for the migration of AA than White TNBC cells, opens up several important avenues of further investigation. An important next step is to determine whether nKIFC1 accumulation is a cause or consequence of tumor aggressiveness. Several studies have implicated KIFC1 in disease aggressiveness and metastases, particularly to the brain^5^. Our data, which establish a tight correlation between KIFC1 nuclear expression and disease outcomes in AA TNBCs, affirms previous reports alluding to a connection between KIFC1 expression and disease aggressiveness^6^. Consequently, our findings make a strong case for examining whether a more direct causative link may exist between nKIFC1 and metastasis, especially given that we uncovered a critical role for KIFC1 in TNBC cell migration. Establishment of a causative role for nKIFC1 in metastasis would position nKIFC1 as a biomarker that prognosticates distant metastasis risk in AA TNBC patients and anti-nKIFC1 targeted therapies as suppressors of further cancer cell dissemination.

nKIFC1 may have distinct functions in different phases of the cell cycle and at different subcellular locations. In mitosis, KIFC1 plays an essential role in the clustering of supernumerary centrosomes in cancer cells^9^. In doing so, KIFC1 not only ensures the survival of cancer cells with amplified centrosomes but may also function as a driver of tumor evolution, as low-grade chromosome missegregation and consequent generation of karyotypic heterogeneity may occur during clustering of amplified centrosomes^16^,^17^. Based on these lines of evidence, the notion that KIFC1 overexpression is responsible for driving evolution of more aggressive phenotypes (e.g., within the group of AA TNBC patients) is plausible. KIFC1 also aids in the focusing of acentrosomal microtubule-organizing centers during the construction of a bipolar mitotic spindle in cancer cells^10^,^18^. Since TNBCs display rampant centrosome amplification^19^, targeting KIFC1 in TNBCs could provide tremendous therapeutic benefits by inducing lethal levels of spindle multipolarity^20^. Most importantly, since KIFC1 is essential for tumor cell viability but is dispensable for the survival of non-cancerous cells^9^, targeting KIFC1 would be an invaluable cancer cell-selective and non-toxic chemotherapeutic approach.

Regarding interphase activities, KIFC1 participates in vesicle transport from the Golgi to the ER in NIH3T3 murine embryonic fibroblasts^21^ along with early endocytic vesicle transport and fission in murine hepatocytes^3^. Our immunohistochemistry data clearly show nuclear accumulation of KIFC1 as a negative prognostic marker in AA TNBCs, suggesting a possible interphase-specific nuclear role for this protein, although perhaps only within this specific patient population. In previous studies, KIFC1 has been implicated in the transport of DNA along microtubules^4^, has been shown to bind nuclear transport factors such as β-importin^22^ and to harbor a specific affinity for nucleoporins NUP50 and NUP153^4^ and a complex containing NUP62^23^. However, the primary sequence of this fascinating protein does not reveal the presence of any recognizable DNA-binding domains. Thus, it is currently unclear what function, if any, KIFC1 serves in the interphase nucleus that might contribute to its ability to promote metastases.

Given the recent progress made in the development of KIFC1 inhibitors^18^, it is also important to identify the patient population that might most benefit from KIFC1-targeted therapy. Pre-clinical development of KIFC1 inhibitors may now benefit from consideration of race and triple-negative status when choosing a disease model. For instance, initial mechanistic study of the first KIFC1 inhibitor implemented a TNBC cell line (BT-549), but it was derived from a White patient. Future analyses of drug efficacy may find other models more favorable (e.g., MDA-MB-468 cells, which are both triple-negative and derived from an AA patient).

## Materials and Methods

### Specimen procurement and datasets

We pooled 163 AA and 144 White TNBC TMAs from multiple institutions, which has been shown to synergistically augment the ability of biomarkers to classify patients into risk groups^24^. Patients were treated between the years 2003–2008 at Grady Memorial and Emory University Hospitals, 2005–2013 at Northside Hospital (all in Atlanta, GA) and 2001–2012 at Scott and White Medical Center (Temple, TX), whose respective Institutional Review Boards approved all aspects of the study. Patient consent was not required due to the archival nature of the de-identified samples. ER, PR, and HER2 staining and scoring were performed as to the prevalent ASCO/CAP guidelines at the time of sample collection. Specimen procurement was also commensurate with the guidelines in effect at the time of collection. Pathologic characteristics were reviewed by the pathologists of the respective hospitals. Descriptive statistics for continuous and categorical variables, including patient and pathologic characteristics, are available in Supplementary Tables 1 and 2, respectively. The median follow up-time was 4.1 years.

### Immunohistochemistry and specimen scoring

TMAs were deparaffinized, rehydrated in serial ethanol baths (100%, 90%, 75%, 50%) and then placed in citrate buffer (pH 6.0) in a pressure cooker at 15 psi for 30 min for antigen retrieval. nKIFC1 was immunostained at 1:1000 dilution in the corresponding author’s lab using rabbit polyclonal antibody, which was graciously provided by Dr. Claire Walczak (Indiana University)^25^. Enzymatic detection of the antibody was performed using the Universal LSAB2 Kit/HRP, Rabbit/Mouse (Dako, CA, US). A panel of potential breast cancer biomarkers (Androgen Receptor; Cytokeratins 5, 7, 8, 14, 18, and 19; CD44, c-Kit; Epidermal Growth Factor Receptor; Insulin-like Growth Factor Receptor; Ki67; Melatonin Receptor; p53; p63; P-cadherin; Survivin [nuclear]; Topoisomerase; Urokinase Receptor [nuclear]; Vimentin; and Zeb) had been previously immunostained^26^. Biomarkers were centrally reviewed and scored by the same two independent pathologists who were blind to clinical annotation. Scoring was performed for both the intensity of staining (0 = none, 1 = low, 2 = moderate, 3 = high) and the percentage of cells with any positivity (i.e., staining of 1+), and the average of the two pathologists’ scores were taken. WIs were calculated as the product of the staining intensity and percentage of positive cells. Representative images of nKIFC1 staining are provided in Fig. 1.

### Statistical analysis of clinical data

Because the distribution of nKIFC1 was strongly right-skewed for both races, non-parametric tests were employed: the Wilcoxon-Mann-Whitney or Kruskal-Wallis test for categorical covariates (with post-hoc pairwise comparisons made using the Dunn-Bonferroni approach) and Spearman correlation for numerical covariates. Monte-Carlo simulations (10,000 samples, 99% confidence interval [CI]) were performed to more robustly estimate all population parameters. Simple and multivariable Cox models of OS, PFS, and DMFS were also fit with bootstrapping (1,000 samples, 95% CI). Plots of partial residuals against rank time were approximately horizontal and centered near zero, indicating satisfaction of the proportional hazards assumption. OS, PFS, and DMFS were defined, respectively, as the number of days from diagnosis to death, death or progression, and death or distant metastasis, whichever occurred first, or last follow-up if no event was recorded. The mean and +/−1 standard deviation (SD) from the mean were tested as cutpoints to stratify patients based on OS. 1 SD above the mean demonstrated a non-significant trend among AA TNBCs, so that cutpoint was used. No trends were noted among White TNBCs, so the AA cutpoint was used for the purpose of comparison. For multivariable Cox models, covariates included AJCC stage, age at diagnosis, adjuvant chemotherapy, and hospital. Nottingham grade, which was not significant in full models, was not included due to multicollinearity with other covariates. The Bonferroni method was used to correct p-values following multiple hypothesis testing. Kaplan-Meier curves were also fit. Statistical analyses were performed using SPSS Version 21 (IBM). All relevant tests were 2-tailed and p < 0.05 was considered significant.

### Statistical analysis of experimental data

Statistical bar graphs with the mean and error bars were plotted using Prism (GraphPad Software Inc., La Jolla, CA, USA). Experimental groups were compared with controls using unpaired two-tailed Student’s t-tests, and p-values were calculated using Prism. p < 0.05 was considered statistically significant.

### Cell culture

White (HCC1143 and MDA-MB-231) and African American (HCC1906 and MDA-MB-468) cell lines were purchased from ATCC. MDA-MB-231 and MDA-MB-468 cells were cultured in Leibovitz’s L-15 medium with 10% fetal bovine serum (ATCC) at 37 °C in 100% air. HCC1806 and HCC1143 cell lines were cultured in Roswell Park Memorial Institute medium with 10% fetal bovine serum (ATCC) at 37 °C and 5% CO_2_.

### siRNA

RNAi knockdown of KIFC1 was performed using ON-TARGET plus KIFC1 siRNA. Non-targeting control siRNAs were used as controls. siRNA pools were transfected using 5 μg siRNA and 5 μl Dharmafect per well of a 6-well plate (resulting in a final siRNA concentration of 25 nM/well) according to the manufacturer’s protocol. All reagents were purchased from Dharmacon (Lafayette, CO, USA). Efficiency of RNAi knockdown was analyzed after 36 h of transfection by Western blot analysis using antibodies specific to KIFC1. The knockdown efficiency was >90%.

### Western blotting and antibodies

36 h after transfection, cells were scraped from culture plates and sonicated in lysate buffer with 1X protease inhibitor cocktail (Invitrogen). Polyacrylamide gel electrophoresis was performed for protein resolution, and then transfer was made to a polyvinylidene difluoride membrane. The membrane was incubated in 1:1,000 mouse monoclonal antibody specific for KIFC1 (M-63: sc-100947) from Santa Cruz Biotechnology (Dallas, TX, USA) with 1:10,000 goat anti-mouse secondary antibody (Abcam; Cambridge, MA, USA). Β-actin was used as an internal loading control and was detected using rabbit monoclonal anti-Β-actin antibody with goat anti-rabbit secondary antibody (both from Abcam). The Pierce ECL detection kit (Thermo Scientific; Waltham, MA, USA) was used to visualize bands.

### Cell proliferation assay

Cell proliferation assay was performed using the BrdU Cell Proliferation Kit (#2750, EMD Millipore). Equal quantities of cells were seeded into 96-well plates. Following attachment, cells were incubated in BrdU for 4 hrs, and BrdU incorporation was measured spectrophotometrically as per kit instructions using TMB as a substrate. Absorbance was recorded at 450 nm. Each experiment was performed at least in triplicate.

### Wound healing assay

Cells were seeded in 6-well plates and cultured until confluent, then they were serum starved for 2 h prior to the assay. Using a 100 ul pipette tip, a scratch was made keeping the pipette tip under an angle of around 30 degrees to keep the scratch width limited, simulating a wound. After scratching, the cells were washed in 1X Dulbecco’s phosphate-buffered saline, serum-containing medium was added, and cells were incubated for 24 h in 5% CO_2_ at 37 °C. Wound edges were imaged using a 10X objective. All the experiments were performed in triplicate and two values were measured from each scratched region.

## Additional Information

**How to cite this article**: Ogden, A. *et al*. Multi-institutional study of nuclear KIFC1 as a biomarker of poor prognosis in African American women with triple-negative breast cancer. *Sci. Rep.* **7**, 42289; doi: 10.1038/srep42289 (2017).

**Publisher's note:** Springer Nature remains neutral with regard to jurisdictional claims in published maps and institutional affiliations.

## Supplementary Material

Supplementary Material

## Figures and Tables

**Table 1:** Relationships between nuclear KIFC1 weighted index (nKIFC1 WI) and patient and clinicopathological factors.

Categorical Variable	Level	N	nKIFC1 WI Mean Rank	p-value
Nottingham Grade				
White	1	3	17.17	<0.001
	2	25	41.36	
	3	99	71.14	
AA	1	2	78.50	0.21
	2	24	65.46	
	3	135	83.80	
AJCC Stage				
White	I/II	115	62.50	1.00
	III/IV	9	62.44	
AA	I/II	109	75.30	0.02
	III/IV	52	92.94	
Lymph Nodes				
White	Negative	95	61.86	0.82
	Positive	27	60.22	
AA	Negative	96	75.31	0.11
	Positive	63	87.14	
**Continuous Variable**	**N**	**Correlation with nKIFC1 WI (ρ)**	**p-Value**	
Tumor size				
White	127	0.15	0.09	
AA	161	0.13	0.09	
Age at diagnosis				
White	144	−0.10	0.25	
AA	161	−0.08	0.33	

Race was self-reported. AA = African American.

**Table 2:** Impact of nuclear KIFC1 weighted index on survival outcomes.

Outcome	Race	HR	p-value	95% CI	
				Lower	Upper
Simple Cox model					
OS	White	0.56	1.00	0.04	1.97
	AA	1.82	0.30	0.77	3.73
PFS	White	0.85	0.75	0.18	2.16
	AA	1.46	0.29	0.67	2.90
DMFS	White	1.17	0.81	0.04	3.63
	AA	1.55	0.22	0.73	3.02
Multivariable Cox model					
OS	White	0.28	0.15	0.00	1.31
	AA	3.45	0.01	1.40	20.80
PFS	White	0.65	0.50	0.00	2.08
	AA	3.14	0.009	1.07	9.18
DMFS	White	0.73	0.55	0.00	2.76
	AA	3.83	0.007	1.45	16.32

The impact of nuclear KIFC1 weighted index (~1 standard deviation above the mean in both racial groups) on overall survival (OS), progression-free survival (PFS), and distant metastasis-free survival (DMFS) in simple and multivariable Cox models adjusted for AJCC stage, age at diagnosis, adjuvant chemotherapy, and hospital at which treatment was received. Bonferroni-corrected p-values are displayed for simple Cox models of OS, for which multiple hypotheses were tested. AA = African American; HR = Hazard Ratio; CI = Confidence Interval.
