# Supplementary material for: Multi-institutional study of nuclear KIFC1 as a biomarker of poor prognosis in African American women with triple-negative breast cancer
Source: Sci Rep. 2017 Feb 20;7:42289. doi: 10.1038/srep42289 (PMC5316996; doi:10.1038/srep42289)
Supplement: Supplementary Material [file srep42289-s1.pdf]

Supplementary Information

Multi-institutional study of nuclear KIFC1 as a biomarker of poor prognosis in African American women with triple-negative breast cancer

Angela Ogden[1], Chakravarthy Garlapati[1], Xiaoxian (Bill) Li[2], Ravi Chakra Turaga[1], Nikita Wright[1], Shristi Bhattarai[1], Karuna Mittal[1], Ceyda Sönmez Wetherilt[1,2], Uma Krishnamurti[2], Michelle D. Reid[2], Mildred Jones[3], Meenakshi Gupta[4], Remus Osan[5], Sonal Pattni[2], Ansa Riaz[1], Sergey Klimov[1], Arundhati Rao[6], Guilherme Cantuaria[3], Padmashree C. G. Rida[1,7*], Ritu Aneja[1*]

[1]Georgia State University, Department of Biology, Atlanta, GA

[2]Emory University School of Medicine, Department of Pathology, Atlanta, GA

[3]Northside Hospital Cancer Institute, Atlanta, GA

[4]West Georgia Medical Center, Department of Pathology, LaGrange, GA

[5]Georgia State University, Department of Mathematics and Statistics, Atlanta, GA

[6]Baylor Scott & White Medical Center, Department of Pathology, Taylor, TX

[7]Novazoi Theranostics, Rolling Hills Estates, CA

*Corresponding Authors:

Ritu Aneja, Department of Biology, Georgia State University, Atlanta, GA, USA
Email: raneja@gsu.edu; Phone: 404-413-5417; Fax: 404-413-5301

Padmashree C. G. Rida, Department of Biology, Georgia State University, Atlanta, GA, USA; Novazoi Theranostics, Rolling Hills Estates, CA, USA
Email: prida@gsu.edu; Phone: 404-783-7733; Fax: 404-413-5301

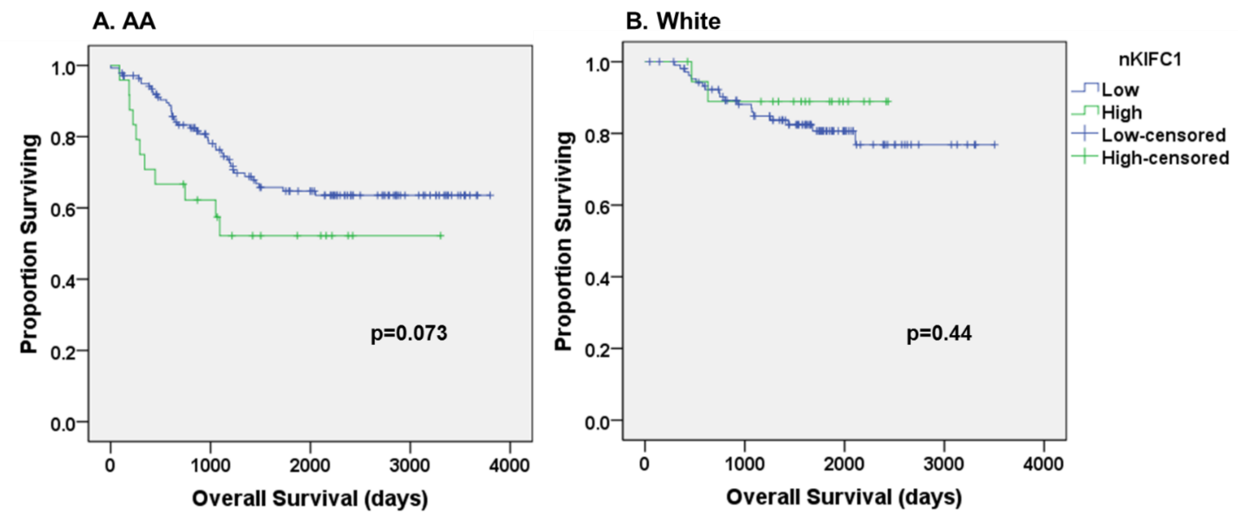

Supplementary Figure 1. Kaplan-Meier curves of overall survival based on nKIFC1 weighted index (stratified by 1 standard deviation above the mean) in **A.** African American (AA) and **B.** White triple-negative breast cancer patients.

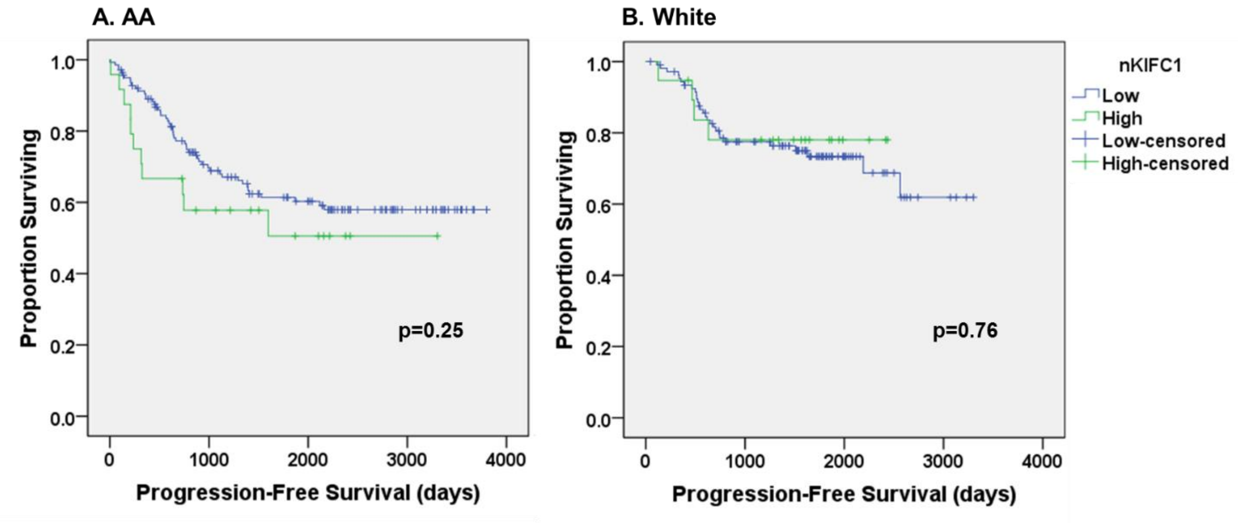

Supplementary Figure 2. Kaplan-Meier curves of progression-free survival based on nKIFC1 weighted index (stratified by 1 standard deviation above the mean) in **A.** African American (AA) and **B.** White triple-negative breast cancer patients.

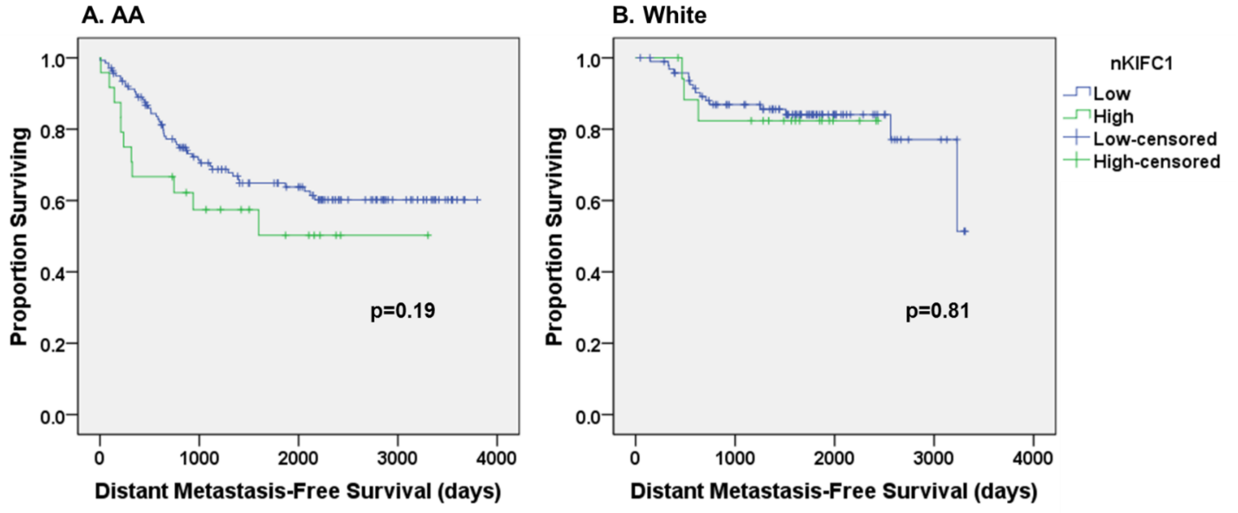

Supplementary Figure 3. Kaplan-Meier curves of distant metastasis-free survival based on nKIFC1 weighted index (stratified by 1 standard deviation above the mean) in **A.** African American (AA) and **B.** White triple-negative breast cancer patients.
